# IInception-CBAM-IBiGRU based fault diagnosis method for asynchronous motors

**DOI:** 10.1038/s41598-024-55367-0

**Published:** 2024-03-02

**Authors:** Zhengting Li, Peiliang Wang, Zeyu yang, Xiangyang Li, Ruining Jia

**Affiliations:** 1 School of Engineering, Huzhou University, Huzhou, 313000 China; 2 Huzhou Key Laboratory of Intelligent Sensing and Optimal Control for Industrial Systems, Huzhou, 313000 China

**Keywords:** Electrical and electronic engineering, Mechanical engineering, Engineering, Electrical and electronic engineering, Mechanical engineering, Engineering

## Abstract

Aiming at the problems of insufficient extraction of asynchronous motor fault features by traditional deep learning algorithms and poor diagnosis of asynchronous motor faults in robust noise environments, this paper proposes an end-to-end fault diagnosis method for asynchronous motors based on IInception-CBAM-IBiGRU. The method first uses a signal-to-grayscale image conversion method to convert one-dimensional vibration signals into two-dimensional images and initially extracts shallow features through two-dimensional convolution; then the Improved Inception (IInception) module is used as a residual block to learning features at different scales with a residual structure, and extracts its important feature information through the Convolutional Block Attention Module (CBAM) to extract important feature information and adjust the weight parameters; then the feature information is input to the Improved Bi-directional Gate Recurrent Unit (IBiGRU) to extract its timing features further; finally, the fault identification is achieved by the SoftMax function. The primary hyperparameters in the model are optimized by the Weighted Mean Of Vectors Algorithm (INFO). The experimental results show that the method is effective in fault diagnosis of asynchronous motors, with an accuracy rate close to 100%, and can still maintain a high accuracy rate under the condition of low noise ratio, with good robustness and generalization ability.

## Introduction

Electric motors are the power source of mechanical operating equipment, of which asynchronous motors account for more than 90%. With their simple structure, high reliability, and easy maintenance, asynchronous motors are widely used in machine tools, power plants, fans, and other industrial fields^1^. As asynchronous motors are often under high load and alternating load conditions, failures are inevitable in the long-term operation process. Once a motor failure occurs in a critical part of the production process, the operation of the equipment may be interrupted, the work efficiency may be reduced, the energy consumption may increase, or the whole system may collapse, resulting in substantial economic losses and casualties^2^. Therefore, the study of fault diagnosis for asynchronous motors has crucial theoretical value and practical significance.

In recent years there has been a rise in the use of deep learning for fault diagnosis. Traditional motor fault diagnosis is usually based on threshold judgment methods. However, asynchronous motors usually operate in complex environments where early fault features are often drowned out by noise so that faults are detected too late, resulting in a lag in repairing motor faults. Compared to machine learning-based methods that require manual extraction of features, which is time-consuming and labor-intensive, and the extracted features are susceptible to subjective factors, deep learning has the advantage of automatically extracting feature information from raw data and learning the intrinsic laws of the data by exploring the association between data and faults to perform fault diagnosis on equipment^3^. One of the deep learning is the convolutional neural network (CNN), a supervised deep learning algorithm with a convolutional structure with good self-learning, fault tolerance, and parallel processing capabilities. G Sakkarvarthi et al.^4^ used a CNN-based technique for crop disease detection and classification with high classification accuracy. Jun Zhang et al.^5^ proposed a fault diagnosis method based on continuous wavelet transform scalar map and multi-scale CNN for bearing fault diagnosis. Xudong Song et al.^6^ proposed a wide convolutional kernel CNN-bearing fault diagnosis method that performs well in terms of good performance in terms of fault diagnosis accuracy and real-time performance.M Elsisi et al.^7^ developed a deep CNN-based industrial internet platform with high accuracy and reliability, which can maintain good robustness under different levels of adversarial attacks. Maoyou Ye et al.^8^ proposed a fault diagnosis method based on variational modal extraction and improved 1D CNN, which can automatically identify different types of faults in rolling bearings. However, the CNN only focuses on spatial features and lacks the characterization of temporal information, and the recognition effect may slip when facing complex fault data^9^. In the processing of temporal signals, two variants of Recurrent Neural Networks (RNN), Long Short-Term Memory (LSTM) and Gated Recurrent Unit (GRU), have good results and can extract temporal features in faults well. Hongming Chen et al.^10^ designed a multi-scale CNN-LSTM neural network model and a deep residual learning model for bearing fault diagnosis, and the proposed model can better extract the fault features hidden in the noisy signal. Long Zhang et al.^11^ proposed a two-pass feature fusion CNN-GRU gearbox fault diagnosis method, which has high accuracy.

The methods mentioned above can only extract local information from the input data and cannot selectively focus on the part of the input that has the most significant impact on the result. Therefore, attention as another aspect of the network has gradually attracted the attention of scholars, where CBAM is a fusion of channel attention and spatial attention, which can effectively enhance the role of the main features and improve model accuracy. At present, not much research has been done on CBAM and neural networks. Wenbo Guo et al.^12^ proposed an LSTM-CNN-CBAM intelligent diagnostic model that can extract fault signal features and identify them accurately, which has strong convergence performance and recognition accuracy and can accurately identify various types of faults. Jun Li et al.^13^ proposed a two-stage attention recurrent neural network and CBAM combined. The results show that incorporating CBAM can effectively improve fault diagnosis accuracy under unbalanced data conditions. Xiaoan Yan et al.^14^ constructed a multi-attention fused residual CNN with a squeeze excitation module and a CBAM module to improve the feature learning performance and achieve automatic identification of mechanical faults.

The above study shows that deep learning methods can effectively detect fault features and diagnose faults in electric motors, but two problems need to be further solved. One is that the traditional deep learning method has limited feature extraction capability and has specific diagnostic errors. The second is that in the actual working process, the motor is always affected by uncertain environmental noise factors, and the traditional deep learning method is not ideal for fault diagnosis in a robust noise environment. Therefore, this paper proposes an end-to-end asynchronous motor fault diagnosis method based on IInception-CBAM-IBiGRU to solve the above problems. The method not only automatically extracts the fault characteristics of asynchronous motors but also effectively avoids the problem of the high computational complexity of deep convolution, which easily produces defects such as overfitting and gradient vanishing and effectively suppresses the influence of strong noise. The main contributions of this study are as follows:

- Improvement based on the Inception module, adding the idea of the residual unit so that it can fully mine the feature information of the data, to alleviate the problem of gradient disappearance brought about by increasing the depth in the deep neural network, and adding a batch normalization layer in the module to avoid overfitting, which effectively improves the fault recognition ability.
- The Dropout layer is embedded on top of BiGRU to extract timing features while preventing the network from overfitting, enhancing the network’s generalization, and improving the fault identification capability.
- A new end-to-end intelligent fault diagnosis model is established by combining IInception, CBAM, and IBiGRU and using INFO to find the optimal network hyperparameters. The model can adaptively find the optimal hyperparameters, automatically learn the input data’s critical spatial and temporal features, and has a high fault diagnosis accuracy.
- The proposed model outperforms the traditional deep learning model by mining out the essential features using residual-structured IInception and CBAM modules and incorporating batch normalization and Dropout to reduce external interference to a certain extent and improve the model’s noise immunity. The model can maintain high accuracy in a low noise ratio environment, with good robustness and generalization ability.

The rest of the paper is organized as follows: Section “Basic methods” introduces the basic theory related to IInception, CBAM, IBiGRU and INFO; Section “IInception-CBAM-IBiGRU fault diagnosis model and optimization” describes the proposed asynchronous motor intelligent fault diagnosis method based on the IInception-CBAM-IBiGRU; In Section “Experimental results and analysis”, the Asynchronous Motor Common Fault (AMCF) dataset^15^ and the Case Western Reserve University (CWRU) bearing dataset^16^ are used to validate the effectiveness of the proposed methodology and to evaluate the model’s immunity to interference at low noise ratios; Finally, Section “Conclusions” draws conclusions.

## Basic methods

### IInception module

**Figure 1:**
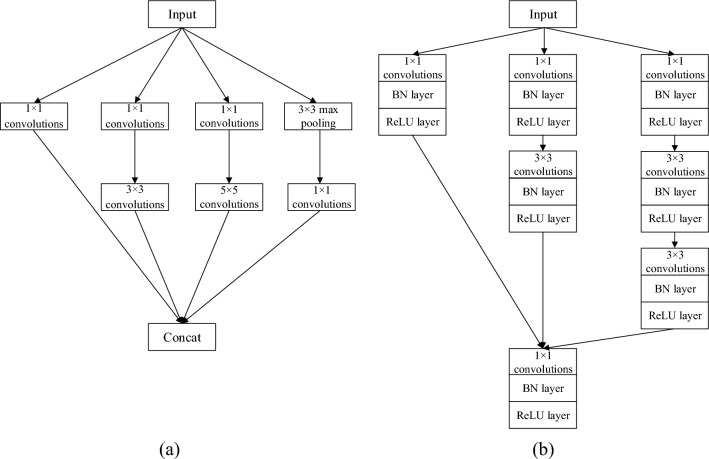
Inception module structure (**a**) Original Inception module structure (**b**) IInception module structure.

In order to maximize the extraction of feature information from the input data, this paper improves the Inception module by introducing a batch normalization layer and a ReLU layer after the convolution layer. The main idea of the Inception module is to use convolutional kernels of different sizes for multi-scale feature extraction, and to increase the network width and improve the network performance by setting multiple channels, while each channel introduces 1 × 1 scale convolutional kernels to downscale the input feature map and reduce the amount of parameter computation, the specific structure is shown in Fig. 1a^17^. Compared with traditional CNN networks, it extracts richer features, improves the adaptability of the network to the scale, and speeds up the network’s training. However, as the depth increases, the network may suffer from the overfitting phenomenon and covariance shift^18^, so this paper introduces the batch normalization layer and ReLU layer after each convolutional layer to enhance the network diagnosis effect, and its structure is shown in Fig. 1b. The batch normalization layer keeps the input data of each layer equally distributed, which not only speeds up the convergence of the network but also has a certain regularisation effect; while the ReLU layer is used to adjust the network output, which enhances the network non-linearity and prevents the model from overfitting; finally, the feature dimensions of different branches are stacked and stitched together, and the operation of dimensionality reduction is achieved by 1 × 1 convolution to reduce the network parameters.

### CBAM module

The attention mechanism can assign different weights to the input features^19^, highlighting the essential features and improving the classification accuracy. This paper uses CBAM attention mechanism to optimize the network for diagnosing asynchronous motor faults. CBAM is a lightweight end-to-end multi-attention mechanism module for convolutional neural networks proposed in 2018, consisting of a channel attention module and a spatial attention module in tandem^20^. Compared to attention mechanisms that only focus on a single aspect, CBAM can focus on both channel and spatial attention, effectively improving the model’s optimization efficiency and prediction accuracy.

**Figure 2:**
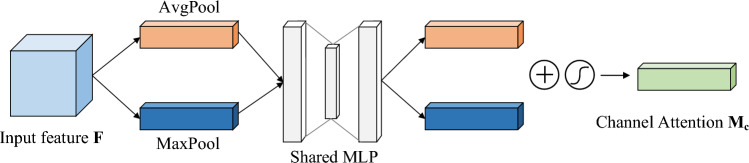
Schematic diagram of the channel attention structure.

The channel attention module is shown in Fig. 2. The channel attention module first performs global max pooling and global average pooling operations on the input features $F$. Then it processes them through the Shared Multi-Layer Perceptron (Shared MLP) model to obtain a mapping of the two-channel attentions. The output features are sequentially summed and activated by the sigmoid function to obtain the channel attention feature weights $M_{c}$, and finally, they are multiplied with the initial features. The output features $F'$ are used as the input features for the spatial attention module. The process of calculating the channel attention module is as follows.

1 $$\begin{aligned} \begin{aligned} M_{c}\left( F\right)&=\sigma \left( MLP\left( AvgPool\left( F\right) \right) +MLP\left( MaxPool\left( F\right) \right) \right) \\ {}&=\sigma \left( W_{1}\left( W_{0}\left( F_{avg}^{c}\right) \right) +W_{1}\left( W_{0}\left( F_{max}^{c}{}\right) \right) \right) \end{aligned} \end{aligned}$$

where $\sigma$ is the sigmoid activation function; MLP is the shared Multi-Layer Perceptron; AvgPool and MaxPool are the average pooling and maximum pooling, respectively; $W_{0}$ and $W_{1}$ are the weight matrices of the MLP; $F_{avg}^{c}$ and $F_{max}^{c}$ are the generated average pooling features and maximum pooling features respectively.

**Figure 3:**
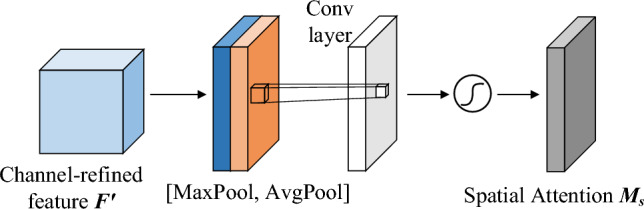
Diagram of the spatial attention structure.

The spatial attention module is shown in Fig. 3. The input features $F'$ are subjected to global average pooling and global maximum pooling operations based on channels, and the resulting features are stitched together with channels, then convolved with a single convolution kernel, and activated with the sigmoid function to obtain the spatial attention feature weights $M_{S}$, and finally inner product operations are performed with the input features of the spatial attention module to generate the final features. The spatial attention module is calculated as follows.

2 $$\begin{aligned} \begin{aligned} M_{s}\left( F^{'}\right)&=\sigma \left( f^{7\times 7}\left( \left[ AvgPool\left( F^{'}\right) ; ( MaxPool\left( F^{'}\right) \right] \right) \right) \\&=\sigma \left( f^{7\times 7}\left( \left[ F_{avg}^{'s};F_{max}^{'s}\right] \right) \right) \end{aligned} \end{aligned}$$

where $f^{7}$ is the 7 × 7 convolution kernel; $F_{avg}^{'s}$ and $F_{max}^{'s}$ are the average pooling feature and the max pooling feature generated, respectively.

### IBiGRU module

**Figure 4:**
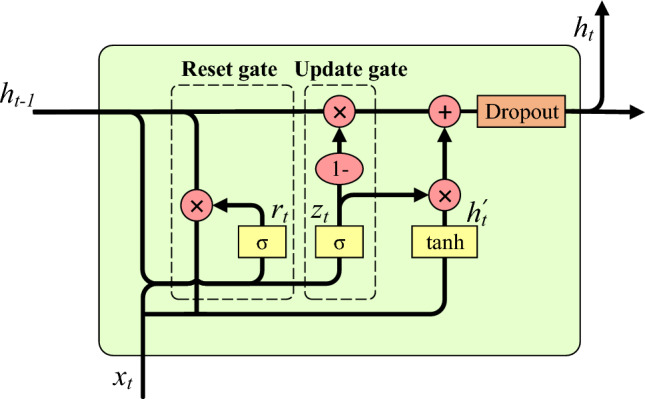
Improved GRU structure.

GRU, as a variant of RNN, can alleviate, to a certain extent, the problem of gradient disappearance during the training process of traditional RNN^21^. Compared with LSTM, which has the problems of complex internal structure and excessive computation due to too many parameters, GRU replaces the input and forgetting gates with update gates on the basis of LSTM structure, reducing the complexity of the structure and the number of parameters, making it converge faster during the training process of the network^22^. However, during the training process, the network inevitably suffers from a certain degree of overfitting, which makes the network more capable of characterizing the training data but less generalizable. Therefore, this paper improves the GRU by embedding a Dropout layer on the output channel of the GRU, stopping some neurons of the network with a certain probability, and reducing the complex relationship between the neurons acting together to alleviate the possible overfitting phenomenon of the model and improve the generalization ability of the model in different scenarios, as shown in Fig. 4. The reset gate state vector $r_{t}$ determines how to combine the previous state information $h_{t-1}$ with the new input information $x_{t}$. The update gate state vector $z_{t}$ indicates how much of the previous moment’s state information, $h_{t-1}$, has been retained. Dropout performs local deactivation of nodes within the output gating parameter matrix, randomly selecting a certain amount of nodes in each training round and setting their hidden parameters to 0 while scaling up the parameters of other non-deactivated nodes accordingly, as shown in Eq. (3).

3 $$\begin{aligned} \begin{aligned} \left. \begin{matrix}r_{t}=\sigma \left( w_{r}\cdot \left[ h_{t-1},x_{t}\right] \right) \\ z_{t}=\sigma \left( w_{z}\cdot \left[ h_{t-1},x_{t}\right] \right) \\ h_{t}^{'}=tanh\left( w_{h}\cdot \left[ r_{t}*h_{t-1},x_{t}\right] \right) \\ h_{t}=\left( \left( 1-z_{t} \right) *h_{t-1}+z_{t}*h_{t}^{'} \right) *m_{drop} \end{matrix}\right\} \end{aligned} \end{aligned}$$

where $\sigma$ and tanh are the sigmoid and hyperbolic tangent functions, respectively; $h^{'}_{t}$ denotes the output candidate after the reset gate process; $x_{t}$ and $h_{t}$ denote the input and output at the current moment respectively; $w_{r}$, $w_{z}$, and $w_{h}$ are the weight matrices of the update gate, reset gate and output pending values respectively; $m_{drop}$ is the dropout probability matrix.

**Figure 5:**
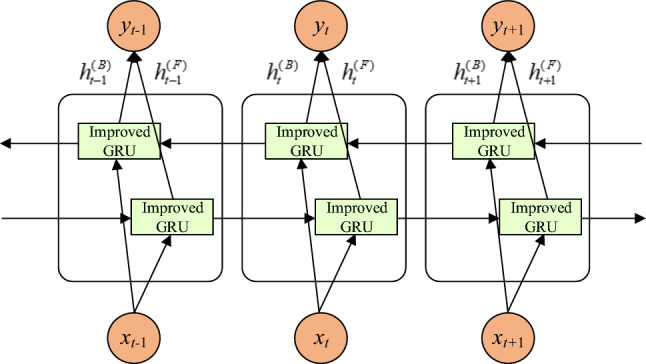
IBiGRU structure.

As the one-way GRU can only link the current input to the previous moment’s information and cannot capture the effect of future inputs on the current input, there are temporal links before and after the fault data of the asynchronous motor. Hence, this paper uses a modified GRU as a unit using the BiGRU model to extract the temporal characteristics of the asynchronous motor fault data, as shown in Fig. 5. IBiGRU combines the advantages of bi-directional RNN and GRU and consists of forward and reverse GRUs stacked up and down, which can simultaneously input sequence data in forward and reverse order. Both forward and reverse GRUs are connected to the output layer, which can pass forward and backward information to the output layer at the same time, establish the connection between the current input and the forward and backward states, and better characterize the timing characteristics of the fault data^21^. The output of IBiGRU, y(t), is expressed as^23^

4 $$\begin{aligned} \begin{aligned} y\left( t\right) =w_{y}\cdot \left[ h^{(F)}_{t},h^{(B)}_{t}\right] +b_{y} \end{aligned} \end{aligned}$$

where $h^{(F)}_{t}$ and $h^{(B)}_{t}$ are the outputs of the forward and backward hidden layers, $w_{y}$ is the weight matrix and $b_{y}$ is the bias term.

### INFO optimization algorithm

The Weighted Mean Of Vectors algorithm is a new population-based intelligent optimization algorithm proposed by Iman Ahmadianfar^24^. Compared with optimization algorithms such as Gravitational Search Algorithm (GSA), Sine Cosine Algorithm (SCA), and Genetic Algorithm (GA), INFO achieves optimization through different vector-weighted averaging rules, which is highly capable of optimizing practical problems in complex unknown search domains with strong optimization ability and fast convergence^24^. The optimization process of INFO is to update the position of the vectors in each generation by updating the rules, merging the vectors, and local searching in three phases.

(1) Update rule phase. A mean-based rule (MeanRule) is extracted from the weighted mean of a set of random vectors to update the position of the vectors, and a convergence acceleration part is added to the update rule operator to improve the global search capability. The specific procedure of the update rule phase is shown in Eq. (5)^25^.

5 $$\begin{aligned} \begin{aligned}{}&z 1_l^g=\left\{ \begin{array}{l}x_l^g+\sigma \times \text{ MeanRule } + \text{ rand } \times \frac{x_{b s}-x_{a 1}^g}{f\left( x_{b s}\right) -f\left( x_{a 1}^g\right) +1}, \text{ rand }<0.5 \\ x_{b s}+\sigma \times \text{ MeanRule } + \text{ randn } \times \frac{x_{a 2}^g-x_{a 3}^g}{f\left( x_{a 2}^g\right) -f\left( x_{a 3}^g\right) +1}, \text{ rand } \ge 0.5\end{array}\right. \\ {}&z 2_l^g=\left\{ \begin{array}{l}x_{b s}+\sigma \times \text{ MeanRule } + \text{ rand } \times \frac{x_{a 1}^g-x_b^g}{f\left( x_{a 1}^g\right) -f\left( x_{a 2}^g\right) +1}, \text{ rand } <0.5 \\ x_{b t}+\sigma \times \text{ MeanRule } + \text{ randn } \times \frac{x_{a 1}^g-x_{a 2}^g}{f\left( x_{a 1}^g\right) -f\left( x_{a 2}^g\right) +1}, \text{ rand } \ge 0.5\end{array}\right. \end{aligned} \end{aligned}$$

where $x_{bs}$, $x_{bt}$ are the optimal and suboptimal vectors in the $g$th generation population, respectively; $\sigma$ is the vector scaling rate; $randn$ is a standard normally distributed random value; $x_{g}^{l,j}={x_{l,1}^{g},x_{l,2}^{g},... ,x_{l,D}^{g}},l=1,2,... ,Np$ is the input vector, where $D$ is the dimension of the vector; $f(x)$ is the value of the objective function; $rand$ is a random value in [0,1]; $z1_{l}^{g}$ and $z2_{l}^{g}$ are the new position vectors of the $g$th iteration.

(2) Vector merging phase. The vectors obtained in the updating rule phase are combined with the vector $x_{l}^{g}$ with condition rand<0.5 to generate the new vector $u_{l}^{g}$ as shown in Eq. (6)^25^.

6 $$\begin{aligned} u_l^g=\left\{ \begin{array}{l} z 1_l^g+\mu . \big |z 1_l^g-z 2_l^g\big |, \text{ rand } 1<0.5 \& \text{ rand } 2<0.5 \\ z 2_l^g+\mu . \big |z 1_l^g-z 2_l^g\big |, \text{ rand } 1<0.5 \& \text{ rand } 2 \ge 0.5 \\ x_l^g, \text{ rand } 1 \ge 0.5 \end{array}\right. \end{aligned}$$

where $\mu$ is $0.05\times randn$, and $u_{l}^{g}$ is the new vector obtained by merging the vectors in the $g$th generation.

(3) Local search phase. This phase effectively prevents the algorithm from falling into local optimal solutions. If rand < 0.5, the vector $u_{l}^{g}$ is generated at the $x_{best}^{g}$ attachment as shown in Eq. (7)^25^.

7 $$\begin{aligned} u_l^g=\left\{ \begin{array}{l}x_{b s}+ \text{ randn } \times \left( \text{ MeanRule } + \text{ randn } \times \left( x_{b s}^g-x_{a 1}^g\right) \right) , \text{ rand } 1<0.5 \& \text{ rand } 2<0.5 \\ x_{r n d}+ \text{ rand } \times \left( \text{ MeanRule } + \text{ randn } \times \left( v_1 \times x_{b s}-v_2 \times x_{r n d}\right) \right) , \text{ rand } 1<0.5 \& \text{ rand } 2 \ge 0.5\end{array}\right. \end{aligned}$$

where $x_{rnd}$ is the new solution consisting of $x_{avg}$, $x_{bt}$, and $x_{bs}$ as shown in Eq. (8), and $v_{1}$ and $v_{2}$ are two random numbers.

8 $$\begin{aligned} \left\{ \begin{aligned} x_{r n d}&=\varnothing \times x_{a v g}+(1-\varnothing ) \times \left( \varnothing \times x_{b t}+(1-\varnothing ) \times x_{b s}\right) \\ x_{a v g}&=\frac{\left( x_a+x_b+x_c\right) }{3}\end{aligned}\right. \end{aligned}$$

where $\phi$ is a random number within (0,1) and $x_{a}$, $x_{b}$, and $x_{c}$ are three random vectors.

## IInception-CBAM-IBiGRU fault diagnosis model and optimization

### IInception-CBAM-IBiGRU model structure

**Figure 6:**
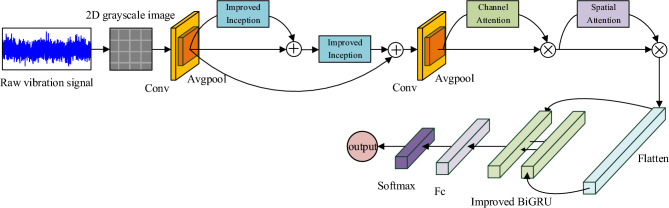
IInception-CBAM-IBiGRU overall structure.

The structure of the IInception-CBAM-IBiGRU fault diagnosis model proposed in this paper is shown in Fig. 6. The model mainly consists of the IInception module, the CBAM module, and the IBiGRU module, where the IInception module is used as a residual block for multi-scale learning and extraction of fault features with a residual structure, the CBAM module further extracts important feature information to achieve optimal fault diagnosis, and the IBiGRU module learns and extracts deep time sequence fault features.

**Figure 7:**
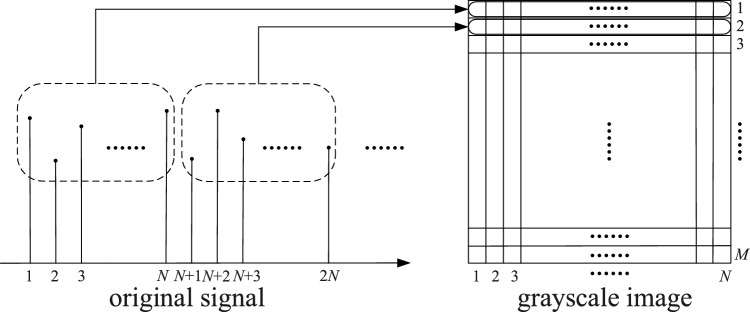
Schematic diagram of signal-to-grayscale image conversion.

To take full advantage of convolution in image classification recognition, a signal-to-grayscale image conversion method^26^ is used to convert a one-dimensional time-series signal into a two-dimensional grayscale map as input, and the conversion process is shown in Fig. 7. First, the amplitude of each sampling point of the input vibration signal is normalized so that it is distributed in the range of 0 to 255 pixels of the image; then, the vibration signal is divided into M sub-intervals, each containing N sampling points, and the values of M and N depend on the total number of sampling points of the vibration signal; finally, the amplitude of the sampling points of each sub-interval is used as the grey scale value of each point in turn, to transform the vibration signal into a greyscale image. This conversion method reflects the essential characteristics of the vibration signal without losing the original data information, and allows the relationship between adjacent sampling points in the signal to be analyzed.

The converted greyscale image is fed into 2D convolution to extract shallow features initially, and the features are downscaled by averaging the pooling layer, and then multi-scale features are extracted. In order to obtain more and more effective fault data information, this paper uses multiple Inception modules to deepen and widen the network structure. However, the end-to-end head-to-tail connection structure of the Inception modules will significantly increase the number of parameters of the model. It cannot effectively use the correlation between the front and back layers, which limits the learning efficiency of the convolution on feature information^27^, and there may also be problems such as gradient disappearance. To address these problems, this paper connects the improved Inception module with a residual structure, effectively reducing the number of parameters and the amount of computation. The jump connection in the residual structure can learn the data information of the previous layer of input, which is conducive to extracting the minute fault features from the data, improving the accuracy and generalization ability of fault diagnosis, and can alleviate the problem of gradient disappearance to a certain extent^28^. Although the residual structure can receive more feature information, given the need for rapid diagnosis of faults, this paper designs two layers of residual networks based on the IInception module to ensure that the whole network is lightweight.

After multi-scale feature extraction, the CBAM module is used to assign higher weights to essential features so that the model focuses on relatively more critical information and reduces the attention to other useless information, improving the efficiency of the model while optimizing and re-adjusting the parameters during the training process to improve the model’s anti-interference capability. The feature map extracted by the CBAM module is then flattened into a one-dimensional vector and fed into the IBiGRU module to extract the different temporal correlations present in the fault signals and to learn the temporal dependencies present in the timing data. The final obtained feature information is fed into the fully connected layer for reintegration, fine-tuning parameters, and mapping to the sample label space. Finally, a Softmax classifier is used to obtain the probability of identifying each fault using the Softmax activation function, taking the maximum value as the model identification result and outputting the diagnosis result.

### INFO optimisation IInception-CBAM-IBiGRU fault diagnostic process

**Figure 8:**
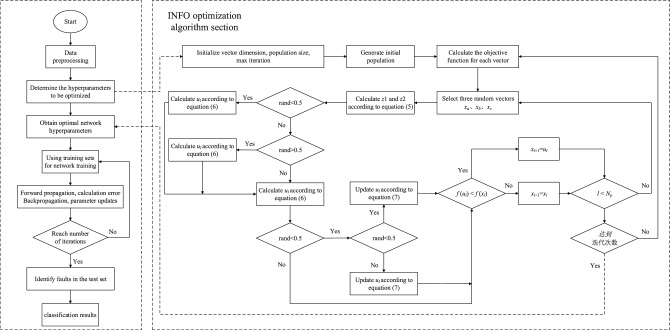
INFO Optimisation IInception-CBAM-IBiGRU flowchart.

INFO is used to optimize the hyperparameters such as learning rate, epoch, and a number of hidden layer nodes in the network model further to improve the fault diagnosis accuracy of the IInception-CBAM-IBiGRU model. Figure 8 shows the flowchart of INFO optimization of IInception-CBAM-IBiGRU hyperparameters with the following steps.

(1) Initialise parameters related to INFO variable dimensions, population size, and number of iterations. Determine the inputs and outputs of the fault diagnosis model.

(2) Calculate each vector’s objective function value $f(x_{i,j}^{g})$ and determine the optimal vector $x_{bs}$.

(3) Calculate the vectors $z1_{l}^{g}$ and $z2_{l}^{g}$ according to Eq. (5) and the vector $u_{l}^{g}$ according to Eq. (6).

(4) Calculate the objective function value $f(u_{i,j}^{g})$ according to Eq. (7), if $f(u_{i,j}^{g})$ < $f(x_{i,j}^{g})$, then $x_{i,j}^{g+1}$=$u_{i,j}^{g}$, otherwise $x_{i,j}^{g+1}$=$x_{i,j}^{g}$.

(5) Judge whether the number of iterations satisfies the termination condition; if not, repeat step (4); otherwise, stop, output the optimal parameters, and get the optimal IInception-CBAM-IBiGRU model for fault diagnosis.

### IInception-CBAM-IBiGRU model parameter settings

The specific parameters of the model, as well as the output feature size and number of each layer, are shown in Table 1. The model adopts the small batch gradient descent algorithm, and the Adam optimizer is used to train the model; the loss function selects the cross-entropy loss, the batch size is set to 128, the number of neurons in the original hidden layer in the IBiGRU is set to 128 and 32, the initial epoch is set to 30, and the initial learning rate is set to 0.001. Hyper-parameters to be optimized: Hidden Layer The number of neurons ranges from [0,300], Dropout ranges from [0.1,0.5], epoch ranges from [20,50], and learning rate ranges from [5e-4,5e-3].

**Table 1:** Parameters of the IInception-CBAM-IBiGRU model.

Layer	FilterSize(Stride)	NumFilters	padding	OutputSize	Activation function
input	–	–	–	1024	–
Preprocessing	–	–	–	32 × 32	–
Conv_1-BN-Activation	3 × 3(1)	32	Same	32 × 32 × 32	eLU
Avgpool_1	2 × 2(2)	-	Same	16 × 16 × 32	–
(IInception Module)					
Conv_2_1-BN-Activation	1 × 1(1)	32	Same	16 × 16 × 32	ReLU
Conv_2_2-BN-Activation	1 × 1(1)/3 × 3(1)	32	Same	16 × 16 × 32	ReLU
Conv_2_3-BN-Activation	1 × 1(1)/3 × 3(1)/3 × 3(1)	32	Same	16 × 16 × 32	ReLU
Depthcat_1	–	–	–	16 × 16 × 96	–
Conv_3-BN-Activation	1 × 1(1)	32	Same	16 × 16 × 32	ReLU
Addition_1	–	–	–	16 × 16 × 32	–
(IInception Module)					
Ibid					
Addition_2	–	–	–	16 × 16 × 32	–
Conv_6-BN-Activation	3 × 3(1)	32	Same	16 × 16 × 32	eLU
Avgpool_6	2 × 2(2)	–	Same	8 × 8 × 32	–
(CBAM Module)					
Channel attention	–	–	–	8 × 8 × 16	–
Spatial attention	–	–	–	8 × 8 × 16	–
Flatten	–	–	–	1024	–
(IBiGRU Module)					
BiGRU_1	–	–	–	–	–
BiGRU_2	–	–	–	–	–
Fc	–	–	–	8	–
Softmax	–	–	–	8	softmax

## Experimental results and analysis

### Experimental data

The experiments in this paper set up data sets A and B. In order to verify that the proposed method can effectively diagnose various types of faults in asynchronous motors, the AMCF asynchronous motor fault dataset^15^ is used as data set A. Considering that bearings are one of the most critical components of asynchronous motors and are also the most prone to failures, fault dataset B adopts the CWRU bearing dataset^16^.

Data set A uses an asynchronous motor model YE2-100L2-4 with a rated power of 3 kW, voltage of 380 V, current of 6.8 A, speed of 1420 rpm, and frequency of 50 Hz. The vibration magnitude in the axial direction (Z-axis direction) at the drive end of the motor is measured by a CT1020 vibration sensor, and the signal is adjusted by a PCH1028 vibration signal monitor, using an acquisition card PCI-1716 to collect the vibration signal with a sampling frequency of 250 kS/s. There are eight motor states in the dataset, one normal state and seven faulty operating states, each with 1000 samples containing 1024 sampling points. Next, the dataset was randomly divided into a training dataset and a test dataset in the ratio of 7:3, with the specific fault types and corresponding labels, as shown in Table 2.

**Table 2:** Experimental dataset.

Label	Type	Fault description	Number of training/testing samples
1	Normal	Healthy	700/300
2	SC2T	Short circuit of 2 turns	700/300
3	SC4T	Short circuit of 4 turns	700/300
4	SC8T	Short circuit of 8 turns	700/300
5	AE	Air-gap eccentricity	700/300
6	RBB	Rotor bar broken	700/300
7	BCB	Bearing cage broken	700/300
8	BAF	Bearing abrasion fault	700/300
Total			5600/2400

The test bench of data set B consists of an asynchronous motor, torque sensor, power test meter, and electronic controller, and the bearing model is selected as SKF6205 deep groove ball bearing with a damage diameter of 0.5334 mm, the signal sampling frequency is 12 kHz, and the window step is set to be 120 so that the one-dimensional bearing vibration acceleration signals at the driving end are sliced into each type of faults by the method of overlapping samples with a sliding window 1000 samples, each sample contains 1024 sample points, and the training and testing data sets are randomly divided according to the ratio of 7:3. The specific fault types and label correspondences are shown in Table 3.

**Table 3:** Experimental data set B.

Label	Type	Fault description	Number of training/testing samples
1	Normal	Healthy	700/300
2	BF	Ball fault	700/300
3	IF	Inner race fault	700/300
4	OF	Outer race fault at center position	700/300
Total			2800/1200

**Figure 9:**
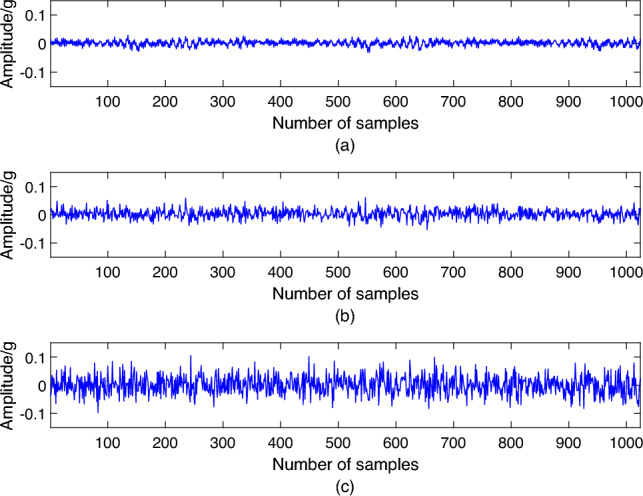
Fault vibration signals.

In a natural industrial environment, the operating environment of a motor is much more complex. Not only is there noise in the working environment but also noise generated by the vibration of its parts and mutual wear, which often pollute the collected vibration signals. Therefore, in order to more realistically simulate the different noise levels in the industrial production environment and better verify the robustness of the model, this paper adds Gaussian white noise with Signal-to-Noise Ratios (SNR) of 2 dB, 0 dB, − 2 dB, − 6 dB and − 10 dB, respectively, into the original data set for the noise immunity experiments. The equation for the SNR is^29^:

9 $$\begin{aligned} \begin{aligned} SNR=10lg\left( \frac{P_{signal}}{P_{noise}}\right) \end{aligned} \end{aligned}$$

where $P_{signal}$ and $P_{noise}$ denote the power of the signal and noise, respectively.

The comparison of adding Gaussian white noise to the vibration signal is shown in Fig. 9. Figure 9a shows the faulty vibration signal without adding noise, and Fig. 9b shows the vibration signal after adding SNR = − 2 dB Gaussian noise. It can be seen that the noise has wholly drowned the original vibration signal. Figure 9c shows the vibration signal after adding SNR = − 10 dB Gaussian noise. As the SNR decreases, the information in the original signal becomes increasingly illegible.

### Comparison of algorithmic optimization

**Figure 10:**
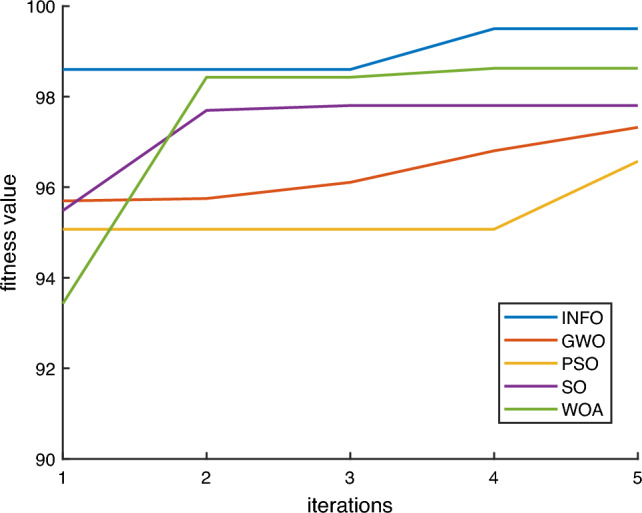
Optimisation iteration curves for each optimization algorithm model.

This paper takes the diagnostic classification accuracy as the fitness function. The hyperparameters of IInception-CBAM-IBiGRU are optimized with INFO, Gray Wolf Optimization (GWO), Particle Swarm Optimization (PSO), Snake Optimizer (SO), and Whale Optimization Algorithm (WOA), respectively. The number of populations is set to 10, and the fitness curves of five optimization algorithms’ models for the optimization iterations are shown in Fig. 10.

Through the comparison in the figure, it can be seen that with the INFO algorithm to optimize the IInception-CBAM-IBiGRU, the first iteration of the fitness reaches 98.60%, which is higher than the other optimization algorithms, and the fitness reaches the optimal value of 99.50% after only four iterations. Compared with other optimization algorithms, the INFO algorithm is faster and more accurate in finding the optimal hyperparameters of the model, and it is highly feasible to use INFO to optimize the hyperparameters of the IInception-CBAM-IBiGRU to establish the fault diagnosis model of the asynchronous motor.

### Analysis of Fault Diagnosis Experiment Results

**Figure 11:**
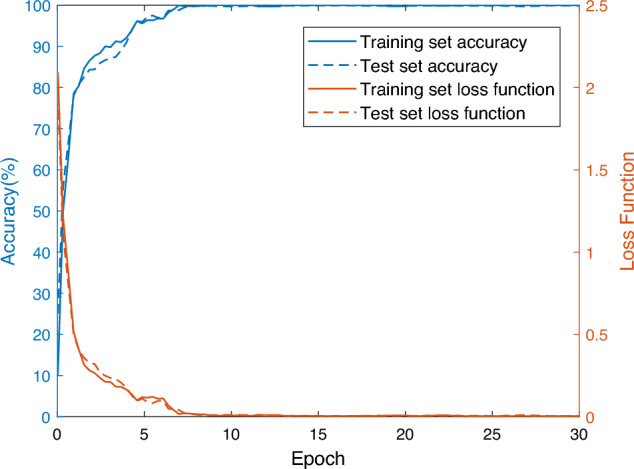
IInception-CBAM-IBiGRU model training and testing process.

In order to verify the effectiveness of the method in this paper, the optimal hyperparameters optimized by the INFO algorithm are input into the IInception-CBAM-IBiGRU for fault diagnosis experiments, and Fig. 11 shows the smoothed curves of the accuracy of the training set and the test set versus the change of the cross-entropy loss value during the model training and testing process. From Fig. 11, it can be seen that the model training process is overall smooth without large fluctuations, and the convergence speed is fast. In the traversal number of 3 times, the accuracy has been more than 90%; when the traversal number reaches eight times, the accuracy and loss function value have been completely converged, and finally, the fault recognition accuracy on the training set reaches 100%, and the loss value tends to be close to 0. In the test set, with the deepening of the model training, the model’s fault recognition accuracy improves rapidly and gradually stabilizes to reach the final 100% accuracy, which indicates that the model of this paper This indicates that the model in this paper has a better training effect, and no severe overfitting and gradient disappearance phenomenon occurs.

**Figure 12:**
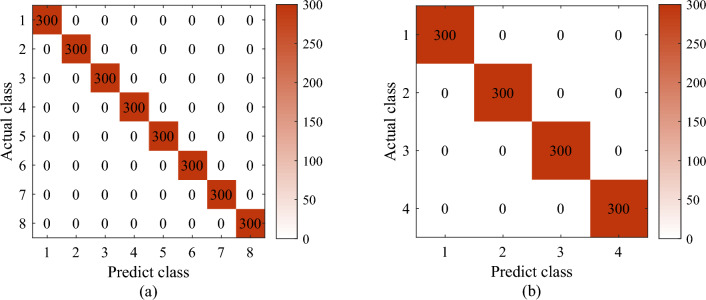
Test confusion matrix diagram (**a**) Test confusion matrix diagram for dataset A (**b**) Test confusion matrix diagram for dataset B.

In order to analyze the effect of the IInception-CBAM-IBiGRU model more intuitively, the diagnostic results are compared with the actual fault types using a confusion matrix, as shown in Fig. 12. The horizontal and vertical axes in the figure indicate predicted and actual fault types, respectively. Observation of the diagonal lines of the confusion matrix shows that each fault type in dataset A and dataset B can be correctly diagnosed, with a fault diagnosis accuracy of 100% and precision, recall, and F1 values of one.

**Figure 13:**
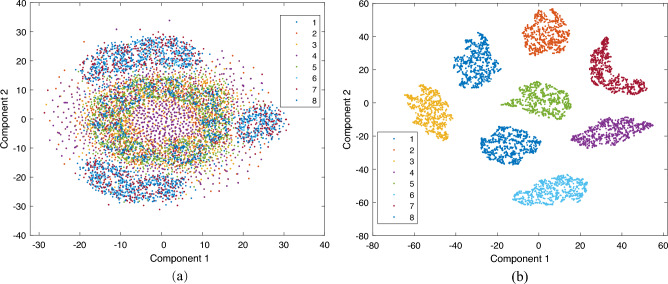
t-SNE dimensionality reduction visualisation (**a**) Initial feature distribution representation of the original data (**b**) Representation of the feature distribution extracted from the output layer of the model.

In order to verify the effectiveness of the method proposed in this paper for asynchronous motor fault feature learning, the t-distributed Stochastic Neighbour Embedding (t-SNE) technique^30^ is used to visualize the features extracted from dataset A, as shown in Fig. 13. The eight colored dots in Fig. 13 indicate the feature distribution of the asynchronous motor under normal and fault conditions, respectively. In Fig. 13a, the eight fault categories of the original dataset are not mixed in any regular way, making it difficult to distinguish between the different fault types of the motors. Figure 13(b) shows that after feature learning of the model, different types of faults can be well separated, and the same types of faults are clustered together, showing better clustering and separability, indicating that the proposed method in this paper has better fault feature learning and extraction capability.

The above results fully demonstrate that the IInception-CBAM-IBiGRU model has fast convergence speed, strong fitting ability, high diagnostic accuracy, and better fault feature extraction ability, and it can diagnose asynchronous motor faults more effectively.

### Analysis of ablation experiment results

In order to verify the reasonableness of the IInception-CBAM-IBiGRU model and explore the influence of each critical component on the overall performance of the model, this paper carries out ablation experiments, adopting dataset A as the data for ablation experiments, removing and comparing each network module of IInception-CBAM-IBiGRU one by one and setting up the same parameters as those of the model in this paper, after ten independent training tests, take the average of the test set results for comparison, the results of the ablation experiments are shown in Tables 4 and 5. Among them, IInception retains the multi-scale residual feature extraction module of this paper’s model, IBiGRU retains the temporal feature extraction module of this paper’s model, IInception-CBAM removes the IBiGRU module based on this paper’s model, IInception-IBiGRU removes the CBAM module, CBAM-IBiGRU removes the IInception module, and Inception-CBAM-BiGRU is the unimproved model.

**Table 4:** Comparison of test results for each model without noise

Model	Accuracy (%)	Precision	Recall	F1-score	Loss
Inception	97.00	0.9386	0.9516	0.9450	0.1066
IInception	98.42	0.9708	0.9836	0.9771	0.0577
BiGRU	92.54	0.9182	0.9650	0.9410	0.2258
IBiGRU	94.12	0.9476	0.9610	0.9542	0.1641
IInception-CBAM	99.71	1	1	1	0.0081
IInception-IBiGRU	99.19	1	1	1	0.0252
CBAM-IBiGRU	95.57	1	1	1	0.1361
Inception-CBAM-BiGRU	99.60	1	1	1	0.0159
Proposed model	100.00	1	1	1	0.0019

**Table 5:** Comparison of test results for each model at each SNR.

Model	2 dB (%)	0 dB (%)	− 2 dB (%)	− 6 dB (%)	− 10 dB (%)
IInception-CBAM	97.69	96.16	95.41	90.76	86.65
IInception-IBiGRU	95.67	94.42	91.92	81.92	79.71
CBAM-IBiGRU	94.17	92.13	84.92	79.29	76.75
Inception-CBAM-BiGRU	96.50	94.75	91.92	83.92	77.75
Proposed Model	98.33	97.67	96.21	92.42	89.83

From Table 4, it can be seen that compared with other models, this paper’s model is optimal in five indicators: accuracy, precision, recall, F1-score, and Loss, where the loss value is much lower than other models, indicating that this paper’s model has the most robust asynchronous motor fault recognition capability. Comparing the results of Inception and IInception and the results of BiGRU and IBiGRU, it can be seen that the improved model in this paper is better than the original model in all the indicators, which indicates that the improved model can show better performance in fault recognition and proves the effectiveness of the improved method in this paper. The comparison results between the unimproved Inception-CBAM-BiGRU model and this paper’s model can also conclude the superiority of the model improvement. Comparing the results of IInception and IBiGRU with those of IInception-IBiGRU yields that the model that considers both spatial and temporal features performs better than the model that considers only a single feature. Comparing the results of IInception and IInception-CBAM with those of IBiGRU and CBAM-IBiGRU, it can be concluded that the CBAM module can further improve the model’s ability to identify faults.

Analyzing Table 5 shows that IInception-CBAM-IBiGRU has the highest model fault identification accuracy under each noise. Comparing the model of IInception-IBiGRU with the model of this paper, it can be seen that CBAM can effectively suppress the influence of noise and irrelevant information on the model. Comparing CBAM-IBiGRU with the model in this paper shows that there is a large gap between the two accuracy rates, reflecting the importance of the IInception module in motor fault diagnosis. Comparing Inception-CBAM and the model of this paper shows that the accuracy difference between the two is not large, which indicates that Inception and CBAM already have better performance, and the introduction of IBiGRU enables the model to learn the timing features, which further stimulates the potential of the model, and effectively improves the fault diagnosis accuracy of the model. Comparing Inception-CBAM-BiGRU with the model in this paper, it can be seen that compared with Inception and BiGRU, IInception, and IBiGRU can better mine the fault feature information from the data in a low SNR environment, effectively improving the model’s anti-jamming ability.

The above results justify the design of the IInception-CBAM-IBiGRU model.

### Analysis of the results of the anti-noise experiment

**Figure 14:**
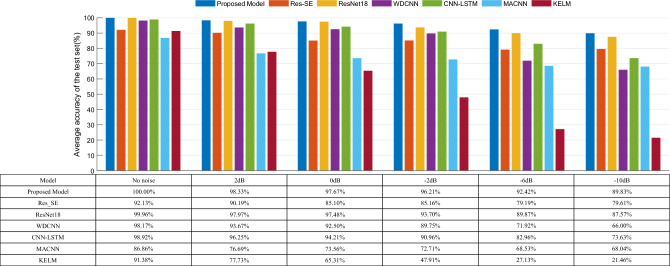
Comparison of the accuracy of each model on the AMCF test set with different SNR.

In order to verify the anti-noise performance of the model in this paper, the dataset A with different SNR signals added is selected for anti-noise experiments, and One-dimensional Dilated Residual Convolutional Networks (Res-SE)^31^, Residual Neural Network (ResNet18)^32^, Wide Convolutional Kernel Deep Convolutional Neural Network (WDCNN)^33^, CNN-LSTM^34^, Multiscale Attention Convolutional Neural Networks (MACNN)^35^, and Kernel-Based Extreme Learning Machine (KELM)^36^ are selected for comparison. Res-SE consists of residual connection blocks, extended residual connection blocks, SE blocks, residual connections, and fully connected layers, which can represent residual Attention Networks.ResNet18 network depth is 18 layers with eight residual blocks and batch normalization layer and pooling layer are added; this model can represent deep residual networks. WDCNN model is a deep CNN with a large convolutional kernel in the first layer and the introduction of the batch normalization processing operation; this model can be used as a representative of a typical deep CNN. The CNN- LSTM model structure includes two layers of convolutional layer, two layers of LSTM, a layer of fully connected layer, and SoftMax function, in which the convolutional layer introduces optimization operations such as batch normalization processing and activation function; this model can be used as a representative of the model combining the CNN and RNN. MACNN consists of three convolutional layers with different scales and channel attention, and it can be used as a representative of the multiscale neural network. The KELM model is an improved model based on the Extreme Learning Machine and combined with the RBF kernel function, which can be used to represent machine learning models. The training and testing rules of each model are the same as above, and the average accuracy of the test set of each model under different SNRs is shown in Fig. 14.

From the histogram in Fig. 14, it can be intuitively seen that each model can achieve better fault recognition accuracy when there is no noise, among which the model in this paper has the highest fault recognition accuracy, indicating that the model in this paper is better at recognizing asynchronous motor faults. As the SNR decreases, except for the model in this paper, the accuracy of the rest of the models shows an apparent decreasing trend, in which the traditional machine learning method KELM is most easily affected by noise, and the accuracy of KELM fault recognition is only about 21% at a low noise ratio of − 10 dB. The WDCNN model, due to the lack of characterization of the temporal features, has a lower accuracy compared to this paper’s model and the CNN-LSTM model. ResNet18 can effectively suppress the effect of network overfitting by using residual connectivity^37^ and has strong fault diagnosis accuracy in noisy environments above − 10 dB, but the model training time is too long due to the superimposition of too many residual modules. The Res-SE and MACNN models are less accurate compared to the models in this paper due to the fact that they only have a channel attention module and lack a spatial attention module, and they also do not take into account timing issues. Therefore, the accuracy is lower compared to the model in this paper. The model in this paper can effectively mine the fault characteristics in the asynchronous motor vibration signals due to the residual structure of the IInception and CBAM modules. At the same time, the addition of batch normalization and Dropout reduces the influence of complex external working conditions to a certain extent. As can be seen from the figure, the accuracy of fault identification in this paper’s model is not significantly affected between the SNR of 2 dB − 2 dB, and the accuracy decreases more slowly after the SNR of − 2 dB, and the accuracy can still be maintained at about 90% even in the case of a very low SNR of − 10 dB. The above results show that the model in this paper has good noise immunity and better robustness than other models.

**Figure 15:**
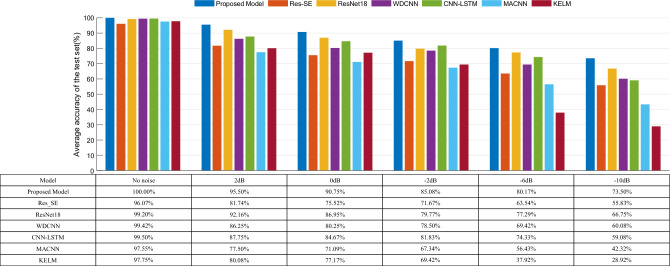
Comparison of the accuracy of each model on the CWRU test set with different SNR.

In order to further verify the robustness of this paper’s model in noisy environments, the above method is used to conduct noise interference experiments on dataset B with noise signals added, and the experimental results are shown in Fig. 15. From the figure can be seen that the accuracy of this paper’s model is higher than that of the other models under the noise interference with different signal-to-noise ratios, which indicates that the Inception-CBAM-IBiGRU still has a good fault diagnosis capability under the noise interference on different datasets.

## Conclusions

In order to improve the model’s ability to extract features from low signal-to-noise signals and make it work in a strong noise environment, this paper proposes an IInception-CBAM-IBiGRU-based fault diagnosis method for asynchronous motors, which allows direct processing of vibration signals for end-to-end fault diagnosis functions. This paper uses the AMCF asynchronous motor fault dataset and CWRU bearing dataset to validate the proposed model experimentally. The results of a large number of comparative experiments show that the improvement of the model in one paper is reasonable. Improving the Inception module and adding the residual module can effectively improve the accuracy of the model fault identification, and the accuracy can be further improved by embedding the Dropout layer in BiGRU and the accuracy of the proposed improvement method is close to 100% for the asynchronous motor fault identification; Second, the model in this paper has superior performance, with a better ability to learn and extract fault features. Between no noise and − 2 dB, the model in this paper can achieve a high fault recognition accuracy. In the low noise ratio between − 6 dB and − 10 dB, the model can still maintain a good level of fault recognition accuracy. It has higher diagnostic accuracy and noise immunity compared to other deep-learning models.

The three-phase asynchronous motor open dataset and the Western Reserve University bearing dataset are used in the current work. Gaussian white noise is added to the data to simulate a noisy environment. The fault situation is a fault state simulated by artificial processing, which is somewhat different compared to faults generated by the actual operation of the motor. In the subsequent research, different types of motor fault data are collected for actual application scenarios further to validate the model’s effectiveness in this paper. In addition, noise has different effects on the diagnostic results of different motor faults, which can be used as a follow-up for further research.

### Supplementary Information

Supplementary Information 1.

## Data Availability

The datasets analyzed during the current study are available in the Zenodo Repository and Case Western Reserve University, The AMCF asynchronous motor fault dataset and CWRU bearing dataset.
